# Outlier Loci and Selection Signatures of Simple Sequence Repeats (SSRs) in Flax (*Linum usitatissimum* L.)

**DOI:** 10.1007/s11105-013-0568-1

**Published:** 2013-02-12

**Authors:** Braulio J. Soto-Cerda, Sylvie Cloutier

**Affiliations:** 1 Cereal Research Centre, Agriculture and Agri-Food Canada, 195 Dafoe Rd, Winnipeg, MB Canada R3T 2M9; 2 Agriaquaculture Nutritional Genomic Center, Genomics and Bioinformatics Unit, CGNA, Km 10 Camino Cajón-Vilcún, INIA, Temuco, Chile

**Keywords:** Adaptive variation, *Linum usitatissimum*, Microsatellites, Neutral variation, Outlier loci, Population structure

## Abstract

Genomic microsatellites (gSSRs) and expressed sequence tag-derived SSRs (EST-SSRs) have gained wide application for elucidating genetic diversity and population structure in plants. Both marker systems are assumed to be selectively neutral when making demographic inferences, but this assumption is rarely tested. In this study, three neutrality tests were assessed for identifying outlier loci among 150 SSRs (85 gSSRs and 65 EST-SSRs) that likely influence estimates of population structure in three differentiated flax sub-populations (*F*_ST_ = 0.19). Moreover, the utility of gSSRs, EST-SSRs, and the combined sets of SSRs was also evaluated in assessing genetic diversity and population structure in flax. Six outlier loci were identified by at least two neutrality tests showing footprints of balancing selection. After removing the outlier loci, the STRUCTURE analysis and the dendrogram topology of EST-SSRs improved. Conversely, gSSRs and combined SSRs results did not change significantly, possibly as a consequence of the higher number of neutral loci assessed. Taken together, the genetic structure analyses established the superiority of gSSRs to determine the genetic relationships among flax accessions, although the combined SSRs produced the best results. Genetic diversity parameters did not differ statistically (*P* > 0.05) between gSSRs and EST-SSRs, an observation partially explained by the similar number of repeat motifs. Our study provides new insights into the ability of gSSRs and EST-SSRs to measure genetic diversity and structure in flax and confirms the importance of testing for the occurrence of outlier loci to properly assess natural and breeding populations, particularly in studies considering only few loci.

**Electronic supplementary material:**

The online version of this article (doi:10.1007/s11105-013-0568-1) contains supplementary material, which is available to authorized users.

## Introduction

Information about the genetic structure of germplasm collections is of great importance for their conservation and utilization (Odong et al. 2011). Several methods have been developed for studying population structure using molecular markers, e.g., *F*_ST_ (Wright 1951), analysis of molecular variance (Excoffier et al. 1992), cluster analysis, principal component analysis, and Bayesian analysis (Pritchard et al. 2000). Although some of these methods have powerful algorithms, the critical factor to take into account remains the type of molecular marker, which should reflect neutral genetic variation to accurately estimate demographic patterns.

Recent advances in DNA sequencing have increased the availability of molecular markers such as genomic (gSSRs) and expressed sequence tag-derived simple sequence repeats (EST-SSRs) facilitating the measurement of genetic variation on a genomic scale (Payseur et al. 2011). Comparisons of both marker types have been assessed to quantify “neutral” genetic diversity in plant in situ populations and ex situ germplasm collections (Wen et al. 2010). In assessing genetic structure with SSR markers, the assumption is that loci are neutral because they are influenced by mutational dynamics and demographic effects and not by selection (Allendorf et al. 2010). However, this assumption is rarely tested before carrying out population genetic studies. Selection affects the genome at specific loci by either reducing the genetic diversity in a specific region in favor of advantageous alleles (positive selection) or by maintaining similar levels of variation across populations (balancing selection) (Wright and Andolfatto 2008). In addition, crop domestication and breeding magnify the occurrence of non-neutral SSR loci of genomic regions underlying agronomic traits. As an effect of genetic hitchhiking, SSR loci closely linked to beneficial alleles might display distortions from neutral expectation. For example, a small proportion (1–5 %) of non-neutral loci can change the estimates of the mean *F*_ST_ values by 30–50 % and also change the topology and branch lengths of dendrograms (Allendorf et al. 2010). As a consequence, non-neutral loci (outliers) could bias estimates of genetic structure and inferences in phylogeographic studies. Nevertheless, outlier loci can better explain the adaptive genetic variation that is not accounted for by neutral loci (Luikart et al. 2003).

Several genome scan studies have been conducted in artificial and natural populations to quantify the percentage of outlier loci and their association to environmental factors. For example, gSSRs have been applied in humans (Storz et al. 2004), sorghum (Casa et al. 2005), and Atlantic herring (Watts et al. 2008) reporting between 4 and 12 % outliers. SSRs identified from ESTs would have a higher probability of detecting the footprints of selection since they occur in coding regions or the sequences that flank them (Rise et al. 2004). Putative EST-SSRs under selection have been identified in maize (Vigouroux et al. 2002), Atlantic salmon (Vasemägi et al. 2005), sorghum (Casa et al. 2006), and three-spined stickleback (Shimada et al. 2011) reporting a range of 1.5–17 % outliers. Since both neutral and non-neutral SSR loci have specific applications in genetic studies, SSR markers accounting for neutral and fitness-related variations must be identified and properly deployed. Furthermore, the potential distorting effect of outlier SSR loci in population structure analysis also needs to be demonstrated in a broader number of species.

Flax (*Linum usitatissimum* L.), a globally important crop because of its seed oil, stem fiber, and functional compounds, is one of the oldest domesticated plants utilized by humans, possibly for as long as 30,000 years (Kvavadze et al. 2009). Recently, the nature of the flax genome has been described through whole genome shotgun sequencing (Wang et al. 2012) complemented by 54 Mb of BAC end sequences (Ragupathy et al. 2011) revealing unique characteristics of its SSRs as compared to other known plant genomes. In the last decade, gSSRs (Wiesner et al. 2001; Roose-Amsaleg et al. 2006; Deng et al. 2010; Soto-Cerda et al. 2011a; Cloutier et al. 2012a) and EST-SSRs (Cloutier et al. 2009, 2012a; Soto-Cerda et al. 2011b) have been developed for flax genetic analyses, including genetic maps (Cloutier et al. 2012b) and QTL studies (Cloutier et al. 2011). However, neither comparisons between gSSRs and EST-SSRs for the assessment of genetic diversity and structure nor the occurrence and effect of outlier SSR loci in population structure have been reported in flax.

In this study, we applied two complementary tests based on the *F*_ST_ parameter as well as the so-called “Schlötterer test” specifically designed for SSR loci (Kauer et al. 2003; Schlötterer 2002) to identify outliers in a set of 85 gSSRs and 65 EST-SSRs using three flax sub-populations. We also compared the ability of these gSSRs and EST-SSRs to measure genetic diversity and reveal population structure before and after removal of outlier loci. The effects, disadvantages, and emerging new applications of outlier loci are discussed.

## Materials and Methods

### Plant Material

A panel of 60 previously characterized flax accessions representing three ancestral sub-populations, namely South Asian (*n* = 10), South American (*n* = 22), and North American (*n* = 28) from 16 countries was included (Soto-Cerda et al. 2012) (Supplementary Table 1). This collection was initially assembled to capture the breadth of seed mucilage variation in flax. The germplasm, representing differing improvement status, including cultivars, breeding material, and landraces, was provided by Plant Gene Resources of Canada and by the Germplasm Bank of the Agricultural Research Institute of Chile INIA.

### Microsatellite Analysis

Genomic DNA samples were extracted from young leaf tissue by the CTAB method (Doyle and Doyle 1987). Eighty-five gSSRs (Roose-Amsaleg et al. 2006; Soto-Cerda et al. 2011a, 2012) and 65 EST-SSRs (Soto-Cerda et al. 2011b, 2012) were evaluated in 60 genotypes as previously described in these references and visualized by silver staining (Bassam et al. 1991).

### Genetic Diversity

Genetic diversity parameters were estimated across the three sub-populations based on the 150 SSR loci and across SSR groups (except allelic richness and private alleles). Allele number, alleles per locus, major allele frequency, expected heterozygosity, and population specific *F*_ST_ were calculated in PowerMarker v.3.25 (Liu and Muse 2005). Allelic richness and the number of private alleles were corrected for sample size differences using the South Asian sub-population as reference (*n* = 10) and estimated using the rarefaction method implemented in HP-RARE v.1.2 (Kalinowski 2005). The polymorphism information content (PIC) was estimated for each sub-population and SSR group (Botstein et al. 1980). Statistical significance for the genetic diversity parameters among sub-populations and SSR groups was estimated by ANOVA and Student’s *t* tests, respectively.

### Detection of Outlier SSR Loci

Since some demographic scenarios such as population structure could increase the number of false positives, the outlier tests were performed for the three previously inferred STRUCTURE sub-populations (Pérez-Figueroa et al. 2010). Two *F*_ST_-based tests (inter-population differentiation) were assessed. The coalescent-based simulation method of Beaumont and Nichols (1996) and the Bayesian test of Foll and Gaggiotti (2008). For the method of Beaumont and Nichols (1996), the program LOSITAN was used (Antao et al. 2008). This method identifies loci under selection based on the joint distributions of expected heterozygosity and *F*_ST_ under an island model of migration. After removing a candidate subset of selected loci (outside the 99 % confidence interval) determined by an initial run with 100,000 simulations, the distribution of neutral *F*_ST_ (depicted by curves around the assessed loci) was computed using putatively neutral loci with 100,000 simulations and a bisection approximation algorithm (Antao et al. 2008). Outliers were identified by comparing observed distribution with neutral expectations at the 99 and 1 % confidence levels (FDR < 0.05). Loci outside the 99 and 1 % confidence areas were identified as candidates affected by positive and balancing selection, respectively. The method of Foll and Gaggiotti (2008) was performed using the program Bayescan 2.0 (http://www-leca.ujf-grenoble.fr/logiciels.htm). The analysis is based on a logistic regression to decompose *F*_ST_ into a *β* component (shared by all loci) and a locus specific *α* component (shared by all the populations). Departure from neutrality at a given locus is assumed when the locus-specific component is necessary to explain the observed pattern of diversity. If *α* > 0, there is indication of positive selection, if *α* < 0, balancing selection is invoked. For each locus, the probability of being under selection is then inferred using the Bayes factor (BF). Based on Jeffreys’ (1961) scale of evidence, a log_10_ BF of 1.5–2.0 is interpreted as “strong evidence” of selection. For our analysis, the estimation of model parameters was set as 10 pilot runs of 5,000 iterations each, followed by 100,000 iterations (Foll and Gaggiotti 2008). Ten independent runs for each method were performed. Only those SSR loci consistently identified as outliers across the 10 independent runs were considered.

The third method, called the ln RH test, identifies loci that differ in variability from the remainder of the genome by calculating the natural logarithm (ln) ratio of gene diversity [(1 / (1 – *H*_pop1_))^2^ − 1] / [(1 / (1 − *H*_pop2_))^2^ − 1] in two populations, where *H* is the expected heterozygosity (Kauer et al. 2003; Schlötterer 2002). Under neutrality, the ln RH is approximately normally distributed (Kauer et al. 2003). Therefore, after standardization of ln RH estimates (mean = 0, SD = 1), 95 % of the neutral loci are expected to have values ranging from −1.96 to 1.96. Loci with ln RH values outside these boundaries were considered outliers.

### Bottleneck Analysis of STRUCTURE Sub-populations

Bottlenecks caused by domestication and artificial selection can mimic the effect of selection, and thus, putative outliers could be false positives (Wright and Andolfatto 2008). Because selection is a locus-specific force and bottlenecks affect all loci across the genome, we hypothesized that selection shaped the distribution of genetic diversity at specific loci, which consequently should show differences in genetic variation in comparison to neutral loci. In other words, if outliers are real and, for example, affected by positive selection, a bottleneck test should be significant for outliers only. On the other hand, under a bottleneck effect, both neutral and outlier loci should show a reduction in genetic variation under a mutation-drift model (Mäkinen et al. 2008); thus, outliers are likely to be false positives. To distinguish between these effects, bottleneck analyses were conducted separately for neutral and outlier loci. Deviations from expected heterozygosity using the program BOTTLENECK 1.2.02 were computed with 5,000 coalescent simulations assuming a two-phase mutation model (TPM) as suggested for SSR data (Cornuet and Luikart 1996) and a step-wise mutation model in each sub-population previously identified (Soto-Cerda et al. 2012). The significance of the deviations was determined by Wilcoxon sign-rank tests.

### Homology Search

The likely functions of the outlier candidates were investigated based on similarity comparisons. Nucleotide-nucleotide (BLASTn) and translated queries versus protein database (BLASTx) were used to identify candidate genes and protein function. Queries were conducted against the NCBI-nt and NCBI-nr (http://www.ncbi.nlm.nih.gov/BLAST), RepBase-Green plant repeat (Jurka et al. 2005), and TIGR plant repeat (Ouyang and Buell 2004) (http://plantrepeats.plantbiology.msu.edu/search.html) databases. A cutoff *E*-value < 10^−15^ was used to infer putative homology and assign functional annotation.

### Genetic Structure Assessment

Because the combination of methods based on different assumptions and modeling approaches can provide more comprehensive genetic structure inferences, we applied Bayesian, similarity-based and principal coordinate (PCo) analyses to compare the utility of the gSSRs, EST-SSRs, and combined SSRs in assessing population structure. The Bayesian analysis was carried out using the program STRUCTURE (Pritchard et al. 2000) with a burn-in of 10,000 and 100,000 iterations for *K* populations ranging from 1 to 10 through 30 independent runs for each SSR group. The admixture model with correlated allele’s frequencies was selected. To determine the optimum number of sub-populations, the average of the log-likelihood (lnP(D)) estimates and the ad hoc statistic ∆*k* for each *K* were calculated (Evanno et al. 2005). The membership coefficient estimate (*Q*) for each accession was calculated by averaging the 30 runs of the best *K* for each SSR group. The similarity analysis was based on the Bray–Curtis similarity index to construct UPGMA dendrograms as implemented in the PAleontological STatistics (PAST) software (Hammer et al. 2001). The reliability of the dendrograms topology was evaluated with 10,000 bootstrap replicates. PCoA was performed in a multidimensional space with data standardization using GENALEX v.6.41 with 1,000 permutations (Peakall and Smouse 2006). The quality of the inferred population structure by the gSSRs and EST-SSRs was compared with the combined SSR loci results previously characterized (Soto-Cerda et al. 2012). The analyses described above were carried out both with and without outlier loci in order to quantify their effects on the inference of population structure accounted for by the three SSR groups.

## Results

### Genetic Diversity Between Sub-populations and SSR Groups

Genetic diversity parameters were estimated for the three reference sub-populations previously characterized using 150 SSR loci (Soto-Cerda et al. 2012). The ANOVA analysis indicated significant differences for all parameters evaluated, except for major allele frequency (*P* = 0.0897), which ranged from 0.77 to 0.81(Table 1). The South Asian, South American, and North American sub-populations showed 53, 19, and 12 fixed loci, respectively. The most structured sub-population was the South Asian (*F*_ST_ = 0.30) followed by the North American (*F*_ST_ = 0.19) and the South American (*F*_ST_ = 0.14). The South American and North American sub-populations presented higher allele numbers, alleles per locus, expected heterozygosity, allelic richness, numbers of private alleles, and PIC values than the South Asian sub-population, with the South American sub-population capturing the highest overall diversity (Table 1).

**Table 1:** Genetic diversity analysis of three flax sub-populations identified by STRUCTURE

	Overall	South Asian	South American	North American	*P* value
Allele number	408	275	345	354	0.0001*
Allele/locus	2.72	1.83	2.30	2.36	0.0001*
Major allele frequency	0.75	0.81	0.77	0.78	0.0897 n.s.
Exp. heterozygosity	0.35	0.26	0.32	0.31	0.0198*
Allelic richness^a^	2.71	1.81	2.21	2.03	0.0001*
Private alleles^a^	–	17	41	20	0.0001*
Pop. specific *F*_ST_	0.19	0.30	0.14	0.19	0.0001*
PIC value	0.30	0.21	0.27	0.26	0.0069*

*n*.*s*. nonsignificant

^a^Corrected by population size (*n* = 10)

*P < 0.05, statistical significance was tested by ANOVA

Within the three reference sub-populations, genomic and EST-SSRs amplified 233 and 175 alleles, respectively. The mean number of alleles per locus was 2.70 and 2.73 for gSSRs and EST-SSRs, respectively (*P* = 0.898). The major allele frequency was the same for both SSR groups (0.746; *P* = 0.978). The expected heterozygosity was 0.346 and 0.351 for gSSRs and EST-SSRs, respectively (*P* = 0.855). The mean PIC value was also similar for gSSRs (0.298) and EST-SSRs (0.300) (*P* = 0.837).

### Detection of Outlier SSRs

**Fig. 1:**
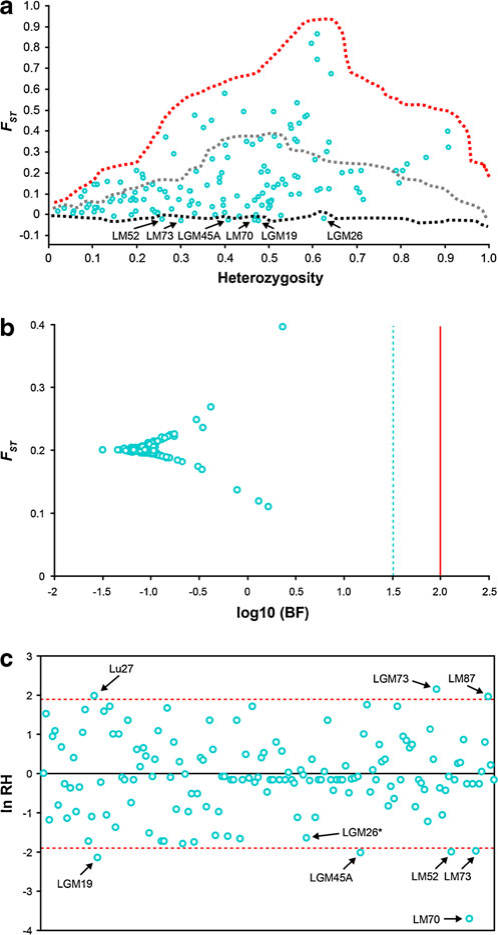
SSR neutrality tests in the three STRUCTURE sub-populations. **a** LOSITAN: distribution of empirical *F*_ST_ values is shown as a function of expected heterozygosity. The *upper* and *lower dashed lines* indicate the 99 and 1 % confidence limits, respectively. The *intermediate dashed line* depicts the median value. **b** Bayescan: distribution of log-transformed Bayes factors and locus specific *F*_ST_. The *dashed* and *solid lines* indicate log_10_ BF of 1.5 and 2 corresponding to posterior probabilities of locus effects of 0.97 and 0.99, respectively. **c** Standardized ln RH: comparison between STRUCTURE sub-populations II and III. *Dashed lines* represent the 95 % confidence interval

Three STRUCTURE sub-populations were used as the references for all posterior comparisons (Soto-Cerda et al. 2012). A global analysis of the three sub-populations was performed to detect putative outlier loci. After constructing the expected distribution of *F*_ST_ in LOSITAN, the overall neutral mean *F*_ST_ was 0.203 ± 0.01. Six loci (4 %) were consistently identified as outliers through 10 independent iterations (FDR < 0.05) including three gSSR loci (LGM26, LGM45A, and LGM19) and three EST-SSR loci (LM52, LM70, and LM73) (Table 2). They appeared in the lower tail of the *F*_ST_ distribution suggesting a signature of balancing selection (Fig. 1a); the remaining 144 loci were considered neutral. For the Bayesian analysis, after 10 independent iterations, no outliers at log_10_ BF of 1.5–2.0 were identified (Fig. 1b). The highest log_10_ BF was 0.36 which, based on Jeffreys’ (1961) scale, corresponds to “barely worth mentioning.” The ln RH test was only conducted between sub-populations II and III because of the larger number of accessions. Sub-population I, with 10 individuals, represents a high risk for false positives associated with its inherent narrower genetic diversity (Table 1). Eight loci were identified outside the confidence boundaries, of which three were suggested to be under positive selection and five under balancing selection (Fig. 1c). The ln RH test confirmed five of the six outlier loci (95 % confidence level) identified by LOSITAN, the exception being LGM26 (ln RH value = −1.87) (Table 2, Fig. 1a). Although two of the three outlier tests were largely consistent, caution should nevertheless be exercised because such outliers could be false positives caused by bottlenecks (see analyses below).

**Table 2:** Candidate SSR outlier loci for balancing selection between three STRUCTURE sub-populations of flax

Locus name	Accession number^a^	Outlier test		BLASTn homology against nt				BLASTx homology against nr			
		*P* value^b^	ln RH^c^	Nucleotide sequence	Identity (%)	*E*-value	Reference	Amino acid sequence	Identity (%)	*E*-value	Reference
LGM19	EU831048	*P* < 0.0001	−2.15	*Populus trichocarpa* predicted protein	72	6*e* ^−85^	XM002320441	*Ricinus communis* conserved hypothetical protein	70	1*e* ^−69^	XP002525312
LGM26	EU830802	*P* < 0.0001	−1.87 n.s.	No hits found	–	–	–	No hits found	–	–	–
LGM45A	EU829744	*P* < 0.001	−2.01	*Populus trichocarpa* EST from severe drought-stressed leaves	84	8*e* ^−22^	CU228355	*Ricinus communis* putative carbonic anhydrase	77	5*e* ^−96^	XP002524642
LM52	EX720477	*P* < 0.001	−2.01	*Populus trichocarpa* chromatin remodeling complex subunit (CHB904)	83	4*e* ^−19^	XM002306612	*Populus trichocarpa* chromatin remodeling complex subunit	59	2*e* ^−68^	XP002306648
LM70	EH791974	*P* < 0.0001	−3.71	No hits found	–	–	–	No hits found	–	–	–
LM73	EH791736	*P* < 0.0001	−1.98	*Xenopus laevis* mRNA for eIF4G-related protein NAT1	100	1*e* ^−35^	AB096099	No hits found	–	–	–

^a^GenBank accession number from which the SSR marker was designed

^b^ *P* value significance determined using LOSITAN (Beaumont and Nichols 1996)

^c^Significant value ln RH calculated according to Kauer et al. (2003). Values between −1.96 and 1.96 are not significant

### Bottleneck Analysis

Because the LGM26 locus was consistently identified by LOSITAN across 10 replications and had an ln RH value close to the confidence limit of −1.96, we included it in the posterior analyses. In order to determine if the outlier loci identified by LOSITAN and the ln RH test correspond to false positives caused by a bottleneck in the STRUCTURE sub-populations, two bottleneck tests were applied. In the analysis of the 144 putative neutral loci, the sign test and the Wilcoxon’s test for heterozygosity excess were significant under the TPM model suggesting that the three STRUCTURE sub-populations have experienced a bottleneck (Table 3). Based on the SSM model, only sub-population I showed signatures of a bottleneck effect. This result could be explained by the small number of accessions (*n* = 10) and its reduced overall genetic diversity (Table 1). With fewer than 20 loci, as is the case of the candidate balancing loci, the Wilcoxon’s test is the most powerful (Piry et al. 1999). For the two mutation model, neither the Wilcoxon’s nor the sign test showed statistically significant bottleneck signatures for the outlier loci in the three sub-populations (Table 3). It is therefore likely that the populations have not experienced reductions in population size, at least in this set of six loci, but other evolutionary forces such as balancing selection might have shaped the pattern of genetic diversity in the candidate regions.

**Table 3:** Bottleneck analysis for the putative neutral and balancing SSR loci in flax

	Neutral (144 loci)				Balancing (6 loci)			
	Sign test		One-tailed Wilcoxon		Sign test		One-tailed Wilcoxon	
	SSM	TPM	SSM	TPM	SSM	TPM	SSM	TPM
Sub-population I (10)	1 × 10^−3^**	3 × 10^−4^***	1 × 10^−5^***	2.6 × 10^−4^***	0.340	0.273	0.093	0.093
Sub-population II (18)	0.177	2 × 10^−3^**	0.107	1.7 × 10^−4^***	0.671	0.643	0.562	0.562
Sub-population III (32)	0.299	3.2 × 10^−4^***	0.310	2 × 10^−3^**	0.570	0.610	0.953	0.921

Sign test and one-tailed Wilcoxon values are *P* values for heterozygosity excess. The analysis was performed for two models of mutation, step-wise mutation model (SMM) and two-phase mutation model (TPM). Numbers in brackets represent the number of accessions per STRUCTURE sub-population

**P < 0.01; ***P < 0.001

### Homology and Putative Function of Outlier Loci

Homology search was used to assign a putative function to four of the six outlier loci analyzed (Table 2). Identity at the nucleotide and amino acid levels varied from 72 to 100 % and 59 to 77 %, respectively. Only the best hits are shown. Five outliers had a trinucleotide motif with the exception being LGM26 which had a tetranucleotide motif (Supplementary Table 2). Four outliers were located within open reading frame sequences, one within the three prime untranslated region (3′UTR) and one remained unknown because the expressed sequence from which it was derived did not produce any significant match against the NCBI nr database (Supplementary Table 2).

### Genetic Structure Assessment

**Fig. 2:**
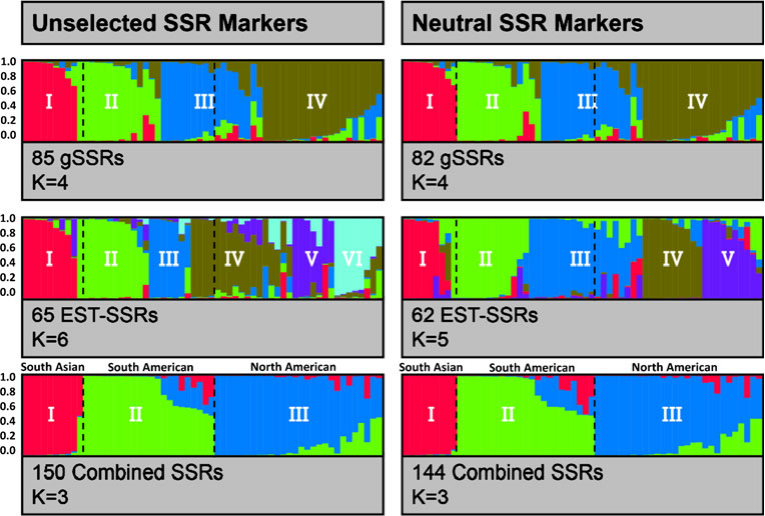
Estimation of the hypothetical ancestral groups for 60 flax accessions based on gSSRs, EST-SSRs, and combined SSRs using STRUCTURE with (*left panel*) and without (*right panel*) the six outlier loci. Each individual is represented by a vertical column partitioned into *K* colored segments proportional to their ancestry probability to each sub-population. *Dashed lines* indicate the reference sub-populations identified by combined SSRs

Using the model-based Bayesian analysis for estimating the number of sub-populations (*K*), the lnP(D) increased with increasing value of *K* but did not show evidence of a maximum for any of the SSR classes (Supplementary Fig. 1). The ad hoc measure Δ*K* showed the highest likelihood at *K* = 4, *K* = 6, and *K* = 3 for gSSRs, EST-SSRs, and combined SSRs, respectively (Fig. 2, Supplementary Fig. 1). The sub-population I identified by the three SSR classes was similar including accessions of Indian and Pakistani origin. The sub-population II showed similarities across the three SSR groups clustering accessions of South American origin but EST-SSRs differed from the others including accessions of Canadian origin too. The sub-population III showed similarities between gSSRs and combined SSRs including accessions predominantly from North America and Europe but EST-SSRs clustered only genotypes from Argentina and Uruguay. The additional sub-populations identified by gSSRs and EST-SSRs followed to some extent a geographic pattern for a few accessions but a higher level of admixture was found as compared to the combined SSRs. The resolution of the grouping performed by combined SSRs was superior to the separate gSSRs and EST-SSRs clustering the 60 accessions mainly but not essentially according to their geographic origins (Fig. 2, Supplementary Table 1). Overall, the level of biological meaning based on pedigree and geographic origin (Plant Gene Resources of Canada) decreased in the following order: combined SSRs, gSSRs, and EST-SSRs.

After removing the six putative outlier loci, the lnP(D) again increased with increasing value of *K* but did not show evidence of a maximum for the three SSR classes (Supplementary Fig. 1). The ad hoc measure Δ*K* showed the highest likelihood at *K* = 4, *K* = 5, and *K* = 3 for the candidate neutral gSSRs, EST-SSRs, and combined SSRs, respectively (Fig. 2). The Δ*K* analysis for the 62 neutral EST-SSR loci showed improvement with a sharper decrease as compared to the 65 EST-SSR loci (Supplementary Fig. 1). Sub-population I, identified by 62 neutral EST-SSR loci, overlapped with sub-population I of combined unselected SSRs (Fig. 2). Sub-population II was largely consistent for the two groups of EST-SSRs. Sub-population III of neutral EST-SSRs combined genotypes from sub-populations III and IV identified by the unselected EST-SSRs. In general, the 62 neutral EST-SSRs performed better in terms of the *K* sub-populations identified, level of admixture, and behavior of Δ*K* analysis after removing the three candidate outlier EST-SSRs. However, the biological meaning of the five sub-populations was still poor compared to the three sub-populations identified by the combined SSRs. Sub-populations identified by the 82 gSSR and 144 putatively neutral SSR loci were consistent to those clustered by 85 gSSR and 150 unselected SSR loci with only minor differences (Fig. 2). Our results showed a “buffer effect” upon the potential bias caused by outlier loci as the number of putative neutral loci increases. The level of biological meaning followed the same order as the unselected groups of SSR loci.

### Similarity Analysis

The UPGMA dendrograms displayed by the three groups of SSR loci revealed different population clustering patterns (Supplementary Fig. 2a, c, e). Based on the 85 gSSR loci, the cophenetic correlation and average bootstrap values were 0.75 and 29.7 %, respectively (Supplementary Table 3) and four main clusters were identified. In contrast, the cophenetic correlation and average bootstrap values for the 65 EST-SSR loci were 0.71 and 24.2 %, respectively (Supplementary Table 3). The overall topology of the UPGMA dendrogram differed from that of the gSSRs but also resulted in four main clusters (Supplementary Fig. 2c). For the 150 combined SSR loci, the cophenetic correlation and average bootstrap values were 0.82 and 37.7 %, respectively (Supplementary Table 3). Three main clusters classified the 60 flax accessions in groups consistent with the STRUCTURE results (Supplementary Fig. 2e). The moderately high cophenetic correlation value for the combined SSR loci indicated little distortion between the dendrogram topology and its original similarity matrix. For the other two sets of SSRs, however, distortion increased as revealed by the higher number of clusters and lower bootstrap support. Overall, the quality of the genetic relationships determined based on pedigree and geographic origin information accounted by the three SSR groups decreased in the same order as observed for the STRUCTURE analysis.

Removal of the putative outlier loci resulted in a cophenetic correlation value of 0.76 and an average bootstrap value of 29.1 % (Supplementary Table 3) for the 82 neutral gSSRs, a not statistically significant difference from the full set of 85 gSSRs (*P* = 0.897, Mann–Whitney *U* test). An improvement was observed in the dendrogram topology rather than in the average bootstrap values accounted for by the 62 putative neutral EST-SSR loci (Supplementary Fig. 2d). This group of SSRs registered a cophenetic correlation value of 0.70 and an average bootstrap value of 24.1 %, also not statistically different from the full set of 65 EST-SSRs (Supplementary Table 3) (*P* = 0.969, Mann–Whitney *U* test). A reduction in sub-clustering was observed, and the topology was similar to that accounted for by the combined SSR loci. Finally, for the 144 combined neutral SSRs, the cophenetic correlation value was 0.82 and the average bootstrap value was 37.7 %, not statistically significant as compared to the 150 combined SSR loci (*P* = 0.987, Mann–Whitney *U* test). The dendrogram topologies and genetic relationships were largely similar between both groups of combined SSR loci. Taken together, these data suggest an improvement in dendrogram topology as a result of using neutral markers for EST-SSRs only. Combined SSRs, whether in whole or neutral sets, resulted in a greater degree of genetic resolution compared to any of the separate sets, likely because of the larger number of neutral data points.

### Principal Coordinate Analysis

Principal coordinates plots, representing the genetic relationships between flax accessions, for the three SSR classes are presented in Supplementary Fig. 3. The first two principal coordinates accounted for 49.6, 43.3, and 48.3 % of the total genetic variance for the 85 genomic, 65 EST, and 150 combined SSR loci, respectively. The 85 gSSR and the combined SSR loci indicated that the sub-population I from the STRUCTURE analysis was the most genetically distant (Supplementary Fig. 3a, e). The 65 EST-SSRs showed a rather scattered distribution of accessions with low discrimination power to separate sub-population I (Supplementary Fig. 3c). The STRUCTURE sub-populations II and III were the most similar with the combined SSR loci showing a better resolution followed by gSSR loci. The principal coordinate plots did not show major differences when the outlier loci were removed. The first two principal coordinates accounted for 49.8, 44.1, and 48.3 % of the total genetic variance for the 82 genomic, 62 EST, and 144 combined neutral SSR loci, respectively (Supplementary Fig. 3b, d, f).

## Discussion

The assessment of genetic diversity is a critical factor for the conservation and the assembly of germplasm collections. Among the three STRUCTURE sub-populations, the overall genetic diversity was narrow. The South American sub-population accounted for the highest proportion of the total diversity, with the South Asian sub-population capturing the lowest, even after correcting for population size (Table 1). Previous studies across different flax collections have also reported low genetic diversity, likely as a consequence of the mating system, limited gene flow, and breeding methods commonly applied in a rather narrow breeding gene pool (Cloutier et al. 2009; Soto-Cerda et al. 2012). As a consequence, we will need to broaden the genetic diversity to conduct successful breeding in flax.

Genomic SSR markers have been used extensively for DNA fingerprinting and genetic diversity studies in plants including flax (Wiesner et al. 2001; Roose-Amsaleg et al. 2006; Deng et al. 2010; Soto-Cerda et al. 2011a). More recently, EST-SSRs, developed from the transcribed regions of the genome, have been used to assess genetic structure in plants (Ellis and Burke 2007). Previous studies suggested that EST-SSRs are less polymorphic than their counterparts because of the greater DNA sequence conservation of transcribed regions (Varshney et al. 2005). In our study, however, we detected similar levels of genetic diversity between the two groups. We found that the difference in the number of repeats between EST-SSRs (mean = 10.01) and gSSRs (mean = 9.16) was not statistically significant (*t* = −0.932; *P* = 0.352) which may be one of the factors that influenced similar levels of genetic diversity detected (Varshney et al. 2005). Positive correlations between number of repeats and polymorphism in gSSRs have been reported in humans and chimpanzees (Trivedi 2004), rice (Singh et al. 2010), and flax (Soto-Cerda et al. 2011a), and the same tendency has been observed in EST-SSRs reported in summer squash (Formisano et al. 2012).

Differences in gene frequency are widely used to draw inferences about population history based on presumed neutral loci. However, selective processes can also affect neutral loci when the latter is in linkage disequilibrium with other loci subjected to selection. Therefore, it is critical to identify loci affected by selection to exclude them from the genetic structure analysis. Most of the studies reporting high frequencies of selected loci employed pairwise population comparisons for outlier tests (e.g., Shimada et al. 2011). Studies using global comparisons typically identified lower percentages of outlier loci (e.g., Nielsen et al. 2009), which could be construed as conservative, but also help to identify either neutral or outlier loci with applications to wider demographic scenarios rather than very specific environmental conditions. We applied three outlier tests based on different algorithms and assumptions to minimize the possibility of selecting false positives (Vasemägi et al. 2005; Shimada et al. 2011). A total of six SSR loci potentially affected by balancing selection were identified. Although it is expected that footprints of selection should be more frequent in EST-SSRs than in gSSRs (Vasemägi et al. 2005), the incidence of outlier loci did not differ between both types of markers (three for each type). Similar results have been reported in salmonid species (Meir et al. 2011; Shimada et al. 2011). Surprisingly, outlier loci affected by positive selection were not consistently identified by the outlier tests. Pérez-Figueroa et al. (2010) carried out a simulation study to compare three alternative *F*_ST_-based outlier programs to detect loci under positive selection. They observed that the most favorable situation for detecting loci under positive selection is that of a low estimated neutral *F*_ST_ distribution (<0.20) as selective loci would tend to show high *F*_ST_ values. In our study, however, the neutral *F*_ST_ distribution was 0.203 implying that this factor could affect the efficiency of LOSITAN and Bayescan in detecting positive selection. On the other hand, under balancing selection, a high neutral *F*_ST_ distribution would be more favorable for detecting selective loci. Bayescan failed also in identifying outliers affected by balancing selection. Comparisons of *F*_ST_ outlier tests indicated that Bayescan has the lowest type I error but also has limited power in detecting balancing selection (Pérez-Figueroa et al. 2010; Narum and Hess 2011). Because the ln RH test is a ratio of variances in gene diversity, it has an identical expectation for all loci independent of the SSR mutation rate and the effective population size (Casa et al. 2005). The results obtained with the ln RH test were largely consistent with those observed with LOSITAN (Table 1). In addition, the weaknesses of the *F*_ST_-based outlier tests mentioned above did not affect the power of the ln RH test to identify loci under positive selection (Fig. 1c). Alternatively, the low cost of the new sequencing technologies allow genotyping of multiple individuals from population samples with array-based or reduced representation sequencing techniques which along with a reference genome sequence can provide a powerful tool for identifying loci under positive selection without the ascertainment bias of *F*_ST_-based outlier tests (Allendorf et al. 2010). Overall, by combining the properties of different outlier tests, it was possible to reduce the percentage of false positives and to strengthen the candidate status of the identified outlier loci (Vasemägi et al. 2005). However, it is still uncertain if undetected loci under positive selection could affect the population structure inferences of the three SSR groups owing to the high neutral *F*_ST_ distribution (0.203) and strong population structure (Table 1).

Population bottlenecks are a concern for outlier analyses (Shimada et al. 2011). Recent studies suggest that current outlier tests underestimate the effects of demography which can create false positive signatures of selection (Excoffier et al. 2009). Nonetheless, BOTTLENECK analyses provided no evidence of recent population bottlenecks for the outlier loci under the TPM and SSM models, and the TPM model was the only test that showed evidence of any loss of genetic diversity for the neutral loci (Table 2). Therefore, it is unlikely that our outlier tests were unduly influenced by the effect of severe population bottlenecks, suggesting that the six outlier SSR loci are true candidates. Balancing selection has been invoked to explain the variability patterns in some gene systems related to the immune response in vertebrates, such as the major histocompatibility complex (Radwan et al. 2010) and the human leukocyte antigen class I (Abi-Rached et al. 2011). In flax, the direct interaction between resistant (R) and avirulent (Avr) proteins is the basis of gene-for-gene specificity in the flax–rust system and both R and Avr genes display signatures of balancing selection (Ellis et al. 2007).

It is important to note that significant deviation from neutral expectations using multiple neutrality tests only raises the candidate status of a particular locus but does not demonstrate selection per se (Vasemägi et al. 2005). Therefore, the identified candidate loci will serve as a basis for further sequence analysis to validate the role of selection in a set of candidate genes. In our study, four candidate outlier loci showed similarity with transcripts from castor bean and poplar at high stringency criteria (*E*-value ≤ *e* ^− 19^) (Table 2) consistent with the taxonomic position of flax, poplar, and castor bean within the Malpighiales order (Wurdack and Davis 2009). For example, the EST-SSR locus LM52 (accession EX720477) showed significant homology to both nucleotide and amino acid sequences of a putative chromatin remodeling complex subunit from poplar (Table 2). Organisms respond to changes in their environments and many such responses are initiated at the level of gene transcription where chromatin influences gene expression (Buck and Lieb 2006). Chromatin remodeling factors regulate access to DNA by moving nucleosomes away from a transcription factor binding site (Varga-Weisz 2010). Recent evidence suggests that, in *Drosophila melanogaster* populations, chromatin remodeling factors may buffer environmental variation by increasing the expression of adaptive genes under a gradient of temperatures and that these chromatin remodeling factors could be affected by balancing selection (Levine et al. 2011). Chromatin remodeling factors are also implicated in the maize (*Zea mays*) response to UV-B radiation (Casati et al. 2008). Maize landraces collected at high altitudes showed high UV-B tolerance by constitutively expressing higher levels of genes predicted to encode chromatin remodeling factors as compared to maize genotypes from temperate zones (Casati et al. 2008). Five out of six outliers had trinucleotide motifs that comprise by far the most common motif for SSRs located within protein-coding domains. Trinucleotide SSRs do not cause frame shifts that can effectively inactivate gene expression or code for shorter protein sequences in the alternative form of repeat motifs. As a consequence, trinucleotide SSRs are more tolerated within coding sequences as compared to other repeat motifs that are selected against and our results are consistent with this evidence. Although this study did not intend to validate the functional status of the candidate outlier loci, we provided evidence through a suite of complementary approaches (outlier tests, bottleneck analyses, and homology search) supporting the candidate status of six SSR loci in order to evaluate their effects in assessing genetic structure in flax. Comprehensive characterization and validation of these loci are beyond the scope of this report.

Genetic structure studies are paramount to the characterization of natural populations and germplasm collections and to assist breeding decisions. An important challenge is the choice of suitable molecular markers that reflect unbiased demographic processes. Genomic SSRs and EST-SSRs have been used and compared for genetic studies in other plant and animal systems ( Wen et al. 2010; Hu et al. 2011) but not in flax where EST-SSRs have only been recently developed (Cloutier et al. 2009, 2012a; Soto-Cerda et al. 2011b). The combined results of STRUCTURE, UPGMA, and PCo analyses showed that gSSRs exhibited a better capacity to assign individuals to their hypothetical ancestral populations (Fig. 2, Supplementary Figs. 2 and 3). In this study, the 65 EST-SSRs revealed a higher number of sub-populations and clusters in the STRUCTURE and UPGMA analyses, and the PCoA plot could only discriminate sub-populations II and III. These discrepancies could be due to differences in targeted DNA regions, genomic coverage rate of the different marker groups (Varshney et al. 2005), or the effect of selection at particular loci. In addition, it is worth noting that the gSSRs amplified 58 more alleles than the EST-SSRs, which is critical to improve resolution when accessions are pedigree-related (Soto-Cerda et al. 2012). STRUCTURE, UPGMA, and PCoA results of the combined SSR analysis provided a more comprehensive picture of the genetic relationship among the flax accessions as compared to the two SSR groups considered separately. It is important to mention that, although the 60 accessions represent 16 countries, they capture a rather narrow genetic diversity (Soto-Cerda et al. 2012). Therefore, in addition to obtaining higher resolution, estimation of genetic structure in germplasm collections with a narrow genetic base could be more efficient with both marker types (Hu et al. 2011).

The exclusion of the outlier loci led to an improvement of the STRUCTURE and UPGMA analyses only when EST-SSRs alone were used. This suggests that either natural or artificial selective pressure could affect these loci or others in linkage disequilibrium with them. Thus, the observed changes in the dendrogram topology could lead to incorrect interpretation of genetic relationships or selection of germplasm for conservation. This collection of flax was primarily assembled aiming to improve the seed mucilage content using association mapping. Unaccounted population structure creates false positive associations between unlinked loci. Biased population structure, for example, as an effect of outlier loci, could also mislead the signal of associations. Simulation studies addressing these potential effects of outliers have not been reported so far. It is likely that because of the smaller number of EST-SSRs, the outlier loci effect was more evident in the STRUCTURE and UPGMA results. On the other hand, increased neutral gSSR and combined SSR loci buffered the flawed genetic structure results. However, the majority of the studies dealing with natural populations have considered no more than 15 presumed neutral loci (Nielsen et al. 2006) which increase the risk of biased estimates of diversity. For example, the exclusion of outlier SSRs from the few assessed in *Gadus morhua* and *Castanopsis eyrei* dramatically changed the previous geographical and adaptive inferences (Nielsen et al. 2006; Shi et al. 2011). These results suggest that many previous estimates of genetic structure in animals and plants using a few loci could be biased and that combined tests of neutrality should be carried out to validate the status of the molecular markers (Luikart et al. 2003). On the other hand, neutral markers are uninformative about the adaptive potential of a population. The discipline of population genomics allows the use of outlier loci for inferring adaptive genetic variation or identifying genomic regions influencing economic traits. For example, outlier loci have uncovered the changes of genetic structure in polluted environments in *Pinus sylvestris* and *Fundulus heteroclitus* ( Williams and Oleksiak 2008; Kuchma and Finkeldey 2011). In maize, genome scans of outlier loci have confirmed agronomic and domestication-related quantitative traits previously reported (Vigouroux et al. 2002; Wright et al. 2005). Attempts at predicting heterosis using mostly neutral genetic distance have generally shown low correlation (Flint-Garcia et al. 2009). With the availability of millions of ESTs in public databases, candidate genes carrying SSRs or single nucleotide polymorphisms estimating “adaptive genetic distance” that could better predict heterosis can be identified. To date, population genomics has accounted for at least 21 genome-wide scans for positive selection in humans (Akey 2009), providing new information on the selections that sculpted the human genome (Grossman et al. 2010). Overall, population genomics will contribute to a better understanding of population processes that require neutral and adaptive genetic markers.

Our study provides new insights into the ability of gSSRs and EST-SSRs to assess genetic diversity and structure in flax. Although the effect of outlier loci was not pronounced, our results highlight the importance of testing for their occurrence. Six non-neutral loci were identified and corroborated through multiple approaches showing that their presence in the data set had an effect in the STRUCTURE and UPGMA results of EST-SSRs but not of gSSRs and combined SSRs where the skewed results were compensated by the larger number of neutral loci. With the development of new statistical models and software, the detection of non-neutral loci is now straightforward, and when possible, previous genetic structure studies in plants and animals should be re-examined for the presence and effect of outliers. In conclusion, we predict that the unbiased understanding of the neutral and adaptive genetic structure will be crucial for properly managing natural and breeding populations.

## Electronic supplementary material

Below is the link to the electronic supplementary material.

ESM 1 (DOCX 2502 kb)
