# Supplementary material for: Outlier Loci and Selection Signatures of Simple Sequence Repeats (SSRs) in Flax (Linum usitatissimum L.)
Source: Plant Mol Biol Report. 2013 Feb 12;31(4):978–90. doi: 10.1007/s11105-013-0568-1 (PMC3881565; doi:10.1007/s11105-013-0568-1)
Supplement: Supplementary file 1 — (DOCX 2502 kb) [file 11105_2013_568_MOESM1_ESM.docx]

**Supplementary Table 1** Flax accessions utilized to study outlier loci, population structure and genetic diversity

| Canadian number | Accession name | Code | Origin | STRUCTURE sub-population |
| --- | --- | --- | --- | --- |
| 101540 | Sel Clli-2156(C4) | 1-CAN* | Canada (Breeding material)^a^ | South Asia (I) |
| 101453 | Sel Clli-647(C4) | 2-CAN* | Canada (Breeding material)^a^ | South Asia (I) |
| 101515 | Sel Clli-1987(C4) | 5-CAN* | Canada (Breeding material)^a^ | South Asia (I) |
| 101519 | Sel Clli-1997(C4) | 6-CAN* | Canada (Breeding material)^a^ | South Asia (I) |
| 101466 | Sel Clli-1472(C4) | 8-CAN* | Canada (Breeding material)^a^ | South Asia (I) |
| 97096 | Clli-1995 | 11-PAK | Pakistan (U.I.S.)^a^ | South Asia (I) |
| 101593 | Sel Clli-2697(C4) | 13-CAN* | Canada (Breeding material)^a^ | South Asia (I) |
| 98239 | CIli-1829 | 14-PAK | Pakistan (U.I.S.)^a^ | South Asia (I) |
| 100629 | Murgzani | 34-PAK | Pakistan (Cultivated material)^a^ | South Asia (I) |
| 98254 | Basin | 37-IND | India (U.I.S.)^a^ | South Asia (I) |
| 30860 | Kirovogradskij71 | 7-UKR | Ukraine (Cultivar)^a^ | South America (II) |
| 97153 | Clli-2052 | 10-TUR | Turkey (U.I.S.)^a^ | South America (II) |
| 96846 | Clli-643 | 12-RUS | Russia (U.I.S.)^a^ | South America (II) |
| 33396 | Vera | 26-CHE | Czechoslovakia (Cultivar)^a^ | South America (II) |
| 97459 | CIli-470 | 27-NETH | Netherlands (U.I.S.)^a^ | South America (II) |
| 100763 | Clli-3229 | 29-USA | USA (Breeding material)^a^ | South America (II) |
| 101241 | AP6 | 32-RUS | Russia (Breeding material)^a^ | South America (II) |
| 98100 | CIli-1553 | 38-URU | Uruguay (U.I.S.)^a^ | South America (II) |
| 97361 | CIli-2743 | 39-URU | Uruguay (U.I.S.)^a^ | South America (II) |
| 97623 | Clli-789 | 40-URU | Uruguay (U.I.S.)^a^ | South America (II) |
| 97852 | Clli-1109 | 41-URU | Uruguay (U.I.S.)^a^ | South America (II) |
| 98933 | Wiera | 44-USA | USA (U.I.S.)^a^ | South America (II) |
| IC-3 | Vilcun | 47-CHI | Chile (Landrace)^b^ | South America (II) |
| IC-4 | Rojas-INTA | 48-ARG | Argentina (Cultivar)^b^ | South America (II) |
| IC-5 | Paisano-INTA | 49-ARG | Argentina (Cultivar)^b^ | South America (II) |
| IC-6 | N8-C66 | 50-CHI | Chile (Landrace)^b^ | South America (II) |
| IC-8 | San-C88 | 52-CHI | Chile (Breeding material)^b^ | South America (II) |
| IC-9 | KZ-41 | 53-CHI | Chile (Breeding material)^b^ | South America (II) |
| IC-10 | FC10 | 54-CHI | Chile (Cultivar)^b^ | South America (II) |
| 101195 | Areco-INTA | 55-ARG | Argentina (Cultivar)^b^ | South America (II) |
| 97664 | Malabrigo-INTA | 57-ARG | Argentina (Cultivar)^b^ | South America (II) |
| IC-15 | HLS-3 | 59-CHI | Chile (Landrace)^b^ | South America (II) |
| 96920 | Clli-1416 | 3-TUR | Turkey (U.I.S)^a^ | North America (III) |
| 98192 | CIli-1685 | 4-MOR | Morocco (U.I.S.)^a^ | North America (III) |
| 98753 | CIli-2702 | 9-FRA | France (U.I.S.)^a^ | North America (III) |
| 18975 | AC Carnduff | 15-CAN | Canada (Cultivar)^a^ | North America (III) |
| 18974 | CDC Bethune | 16-CAN | Canada (Cultivar)^a^ | North America (III) |
| 18970 | Flanders | 17-CAN | Canada (Cultivar)^a^ | North America (III) |
| 19002 | Omega | 18-USA | USA (Cultivar)^a^ | North America (III) |
| 72584 | Macbeth | 19-CAN | Canada (Cultivar)^a^ | North America (III) |
| 72582 | Lightning | 20-CAN | Canada (Cultivar)^a^ | North America (III) |
| 33385 | Linott | 21-CAN | Canada (Cultivar)^a^ | North America (III) |
| 44316 | Vimy | 22-CAN | Canada (Cultivar)^a^ | North America (III) |
| 72585 | 84495 | 23-AUS | Australia (Cultivar)^a^ | North America (III) |
| 18973 | AC Watson | 24-CAN | Canada(Breeding material)^a^ | North America (III) |
| 19004 | AC Emerson | 25-CAN | Canada (Cultivar)^a^ | North America (III) |
| 98042 | 10484/46 | 28-ARG | Argentina (U.I.S.)^a^ | North America (III) |
| 100769 | Clli-3246 | 30-USA | USA (Breeding material)^a^ | North America (III) |
| 97520 | Clli-576 | 31-RUS | Russia (U.I.S.)^a^ | North America (III) |
| 101247 | VNIIL-5628 | 33-RUS | Russia (Breeding material)^a^ | North America (III) |
| 100674 | Clli-3026 | 35-ROM | Romania (Cultivated material)^a^ | North America (III) |
| 97415 | Redwing | 36-USA | USA (Cultivar)^a^ | North America (III) |

**Supplementary Table 1** Continued.

| Canadian number | Accession name | Code | Origin | STRUCTURE population |
| --- | --- | --- | --- | --- |
| 98954 | Cascade | 42-USA | USA (U.I.S.)^a^ | North America (III) |
| 98945 | Liral Prince | 43-USA | Canada (U.I.S.)^a^ | North America (III) |
| IC-1 | Ten-C1 | 45-CHI | Chile (Landrace)^b^ | North America (III) |
| IC-2 | Gr-C2 | 46-CHI | Chile (Cultivar)^b^ | North America (III) |
| IC-7 | Sau-C77 | 51-CHI | Chile (Breeding material)^b^ | North America (III) |
| IC-12 | Ent-C112 | 56-ARG | Argentina (Cultivar)^b^ | North America (III) |
| IC-14 | CC-13 | 58-CHI | Canada (Cultivar)^b^ | North America (III) |
| IC-16 | HLS-4 | 60-CHI | Chile (Landrace)^b^ | North America (III) |

*U.I.S*. Uncertain Improvement Status

^a^ Improvement status assigned by Plant Gene Resources of Canada

^b^ Improvement status assigned by Agricultural Research Institute of Chile, INIA-Carillanca (IC number)

* Genotypes selected from either Indian or Pakistani accessions (http://pgrc3.agr.gc.ca)

**Supplementary Table 2** Repeat motif, map and gene location of outlier SSRs

| Locus name | Repeat motif | Linkage group*^a^* | Scaffold*^b^* | Location within gene |
| --- | --- | --- | --- | --- |
| LGM19 | (CCG)_5_ | 7 | 151 | ORF |
| LGM26 | (TTCT)_4_ | 12 | 765 | 3’UTR |
| LGM45A | (TCT)_8_ | 8 | 225 | ORF |
| LM52 | (ACG)_5_ | 10 | 67 | ORF |
| LM70 | (GAA)_6_ | - | 253 | Unknown |
| LM73 | (GAT)_5_ | 13 | 296 | ORF |

*^a^*Map position obtained from the consensus linkage map of flax (Cloutier et al. 2012b)

*^b^*Scaffold position obtained from <http://www.phytozome.net/>

**Supplementary Figure 1**


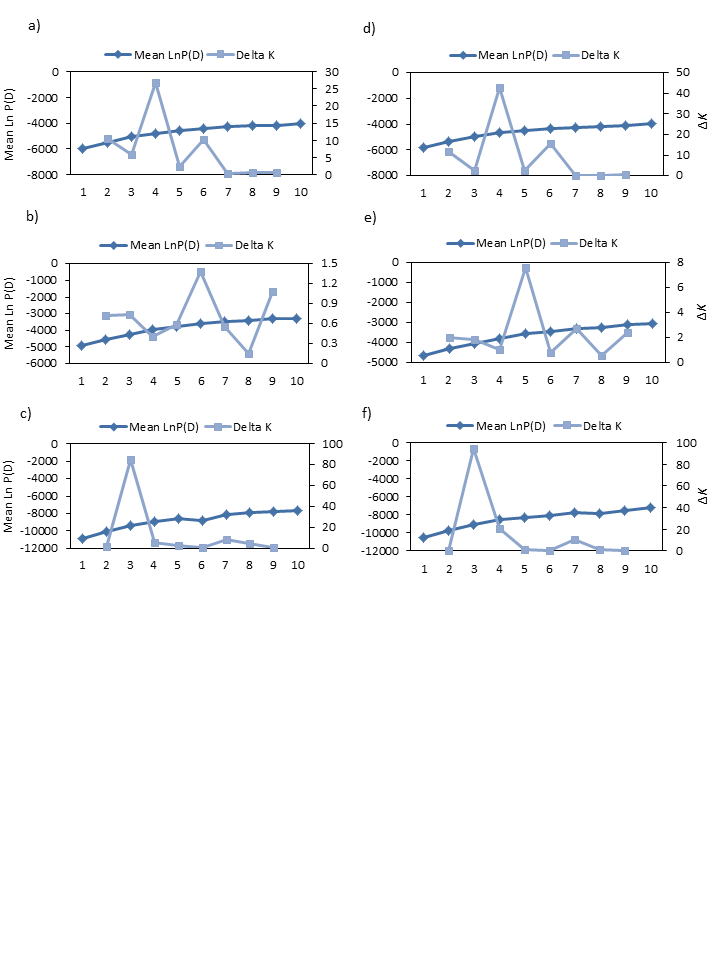


Estimation of the hypothetical number of sub-populations (*K*) using the average *ln* likelihood values (mean Ln P(D) for 30 iterations) and *ad hoc* statistic Δ*K* (Evanno et al. 2005) for *K* values ranging from 1 to 10 using three groups of SSR markers. **a)** 85 gSSRs **b)** 65 EST-SSRs **c)** 150 combined SSRs **d)** 82 neutral gSSRs **e)** 62 neutral EST-SSRs **f)** 144 combined neutral SSRs

**Supplementary Figure 2**


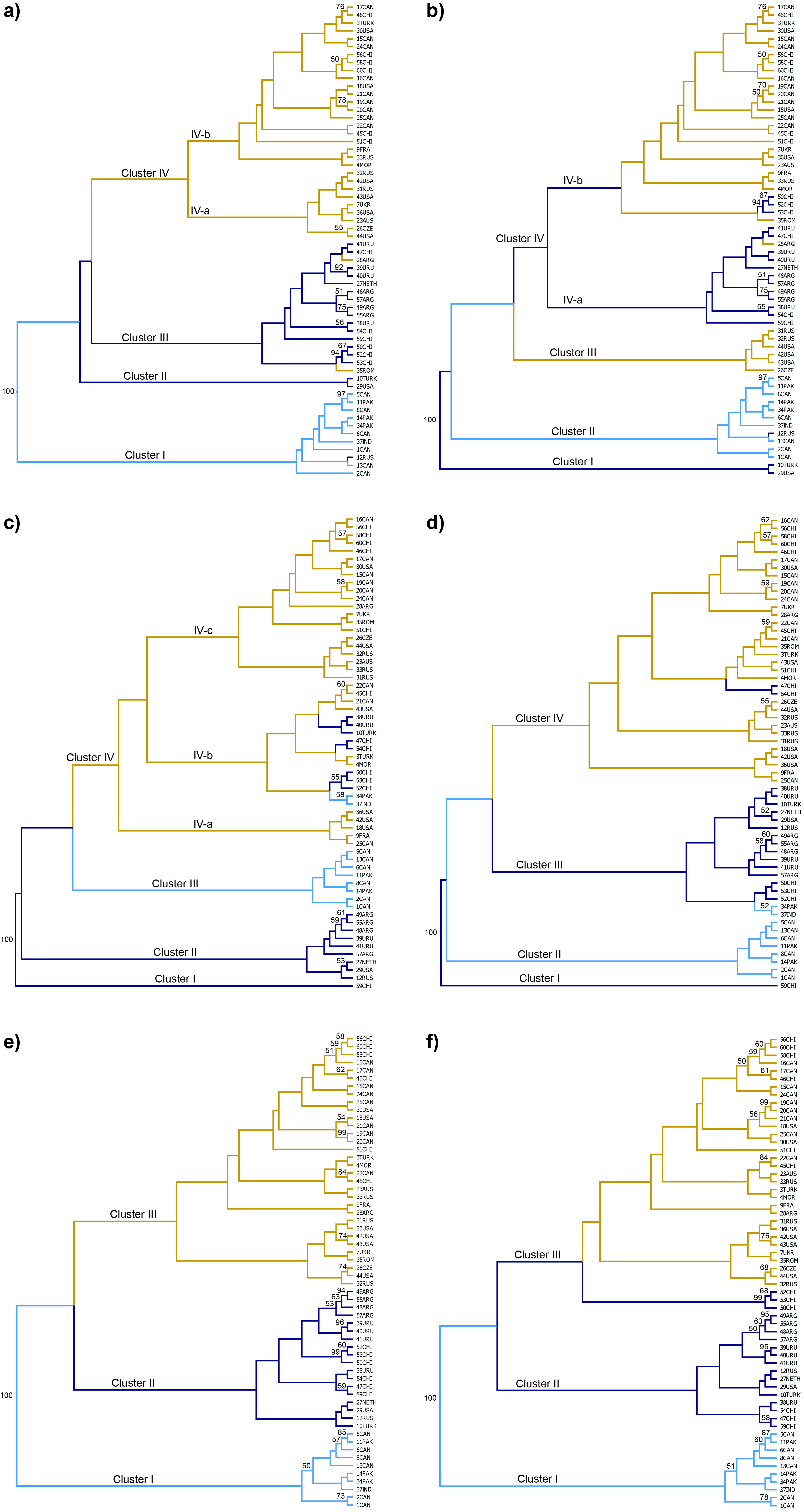


UPGMA dendrograms of 60 flax accessions based on gSSRs, EST-SSRs and combined SSRs before and after removal of the outlier loci. **a**) 85 gSSRs **b**) 82 neutral gSSRs **c**) 65 EST-SSRs **d**) 62 neutral EST-SSRs **e**) 150 combined SSRs (reference dendrograms where Cluster I = South Asian, Cluster II = South American and Cluster III = North American populations) and **f**) 144 neutral combined SSRs. Bootstrap values ≥ 50% are shown (10,000 bootstrap replicates). Color clusters from 150 combined SSRs **e**) were used as references to indicate similar or dissimilar topologies

**Supplementary Table 3** Comparison of bootstrap performance of three SSRs groups

| Marker | Mean bootstrap (%) | % node ≥ 50% | Mean bootstrap of node ≥ 50% |
| --- | --- | --- | --- |
| 85 gSSRs | 29.7 | 20.3 | 74.3 |
| 82 neutral gSSRs | 29.1 | 18.6 | 71.4 |
| 65 EST-SSRs | 24.2 | 15.3 | 62.3 |
| 62 neutral EST-SSRs | 24.1 | 16.7 | 61.4 |
| 150 combined | 37.7 | 35.6 | 71.6 |
| 144 neutral combined | 37.7 | 35.6 | 72.2 |

**Supplementary Figure 3**


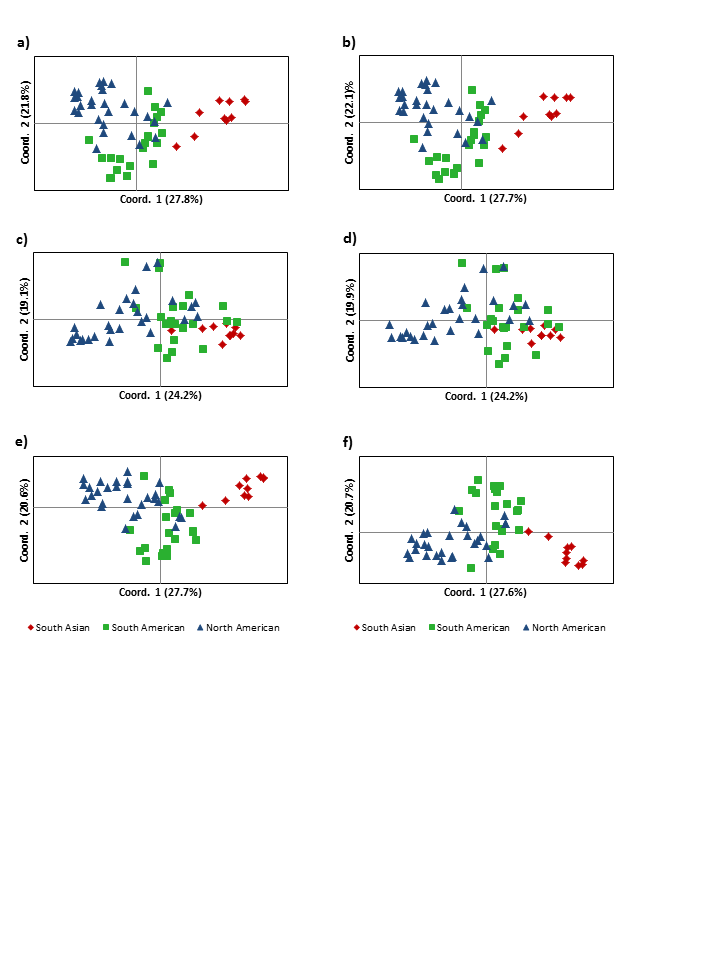


Principal coordinate analysis (PCoA) of three flax sub-populations based on gSSRs, EST-SSRs and combined SSRs before and after removal of the outlier loci. **a**) 85 gSSRs **b**) 82 neutral gSSRs **c**) 65 EST-SSRs **d**) 62 neutral EST-SSRs **e**) 150 combined SSRs and **f**) 144 neutral combined SSRs
